# Clinical efficacy and the antimicrobial potential of silver formulations in arresting dental caries: a systematic review

**DOI:** 10.1186/s12903-020-01133-3

**Published:** 2020-06-03

**Authors:** Kausar Sadia Fakhruddin, Hiroshi Egusa, Hien Chi Ngo, Chamila Panduwawala, Siripen Pesee, Lakshman Perera Samaranayake

**Affiliations:** 1 Department of Preventive and Restorative Dentistry, M28-125, College of Dental Medicine, University of Sharjah, Sharjah, 27272 United Arab Emirates; 2 Division of Molecular and Regenerative Prosthodontics, Tohoku University Graduate School of Dentistry, 4-1 Seiryo-machi, Aoba-ku, Sendai City, 980-8575 Japan; 3 Faculty of Dentistry, Department of Oral Diagnostic Science, Faculty of Dentistry, Thammasat University, Pathum Thani, Thailand; 4 The University of Hong Kong, Hong Kong Special Administrative Region, China

## Abstract

**Background:**

The use of silver-formulation as microbicide to arrest dentinal caries is gaining popularity. The primary objective of the present appraisal was to systematically review the clinical (in vivo) applications and antimicrobial potential of silver-containing formulations in arresting dentinal caries. Our secondary aim was to sum up the available in vitro applications of silver-containing formulations against cariogenic microbes isolated from dentine lesions.

**Methods:**

Ovid MEDLINE, EBSCO host, Web of Science, and Cochrane Library databases was searched between January 2009–May 2019.

**Results:**

In vivo: We observed conflicting evidence of antimicrobial efficacy of SDF on a diverse array of microbial taxa present in carious dentine of primary and permanent teeth. Moreover, there is insufficient evidence on the application of AgNP-fluoride as an effective microbicidal against cariogens of dentine lesions.

In vitro: We found a good evidence of microbicidal efficacy of silver diamine fluoride (SDF) on selective cariogenic microbes in human dentine model. Additionally, a good evidence was noted of in vitro application of silver nanoparticles (AgNPs) as a useful microbicidal against *S. mutans* adhesion, growth and subsequent biofilm formation in human dentine models.

**Conclusions:**

Taken together, in vitro evidence indicates the promising antimicrobial potential of silver-based formulations (SDF and nanosilver) against the predominant cariogenic flora, particularly from dentine lesions. Post-treatment clinical data of either the bactericidal and bacteriostatic effects of SDF or nanosilver are sparse. Furthermore, the current understanding of the specific size, concentration, antimicrobial mechanisms, and toxicological aspects of nano-silver compounds is inadequate to draw firm conclusions on their clinical utility.

## Background

The plaque biofilm microbiota comprising a cariogenic microbiome is the prime mover of both enamel and dentinal caries. Past research provides ample evidence that links the *mutans*-group of streptococci to dental caries [1, 2]. However, a contemporary molecular approach using 16S rRNA gene sequencing and next-generation sequencing (NGS) technology has revealed a diverse spectrum of hitherto undescribed microbiota contributing to the pathogenesis of dentinal caries. For instance, Richards and co-workers [2017] found a high prevalence of *Scardovia wiggsiae*, *Lactobacillus salivarius*, *Streptococcus mutans,* and *Parascardovia denticolens* species, which are all acidogenic, in caries-active dentine lesions in children aged 2–7-years old [3]. Additionally, species belonging to the genera *Actinomyces*, *Bifidobacterium, Veillonella, Selenomonas, Propionibacterium*, and *Atopobium* species are now known to be associated with carious dentine [2, 4, 5].

Relatively recent molecular studies have also uncovered other proteolytic microbes in deep-dentinal caries lesions that may facilitate collagenolysis and subsequent dentine tubular invasion. Of which, gram-negative anaerobe *Prevotella* sp., appears to be the predominant species together with *Propionibacterium* sp., oral strain FMA5, and *Porphyromonas* species, particularly in advanced caries lesions [6–8]. Similar to other proteolytic bacteria, *Candida,* an opportunistic human fungal pathogen, is now considered a secondary perpetrator of the caries process, facilitating dentinal tubular invasion [9, 10].

### Contemporary recommendations for deep-caries management and residual-caries

Present strategies of deep-caries management include selective caries removal, to lessen the risk of pulp exposure and consequent complications, that can compromise the longevity of the tooth [11]. However, up until now, there is no consensus on the quantity of the affected dentine in the carious lesion that should be removed. Clinical criteria indicative of caries-free status after debridement are rather crude and are currently based on naked-eye, subjective assessment of the color of residual caries, hardness on probing, and moisture content of the dentine [12–16].

### Survival kinetics of residual ‘cariogenic’ microbes

Employing contemporary partial removal philosophy, the success related to partial caries removal can occur if a hermetic restoration is placed to avoid microbial proliferation. It is currently surmised that viable microbes entombed within a placed restorations undergo a process of prolonged starvation leading to microbial dormancy and death, thus resulting in lesion arrest [17, 18]. On the contrary, several reports have now described the ability of such entombed microbes are capable of proliferation and survival, even with a limited supply of nutrients [19]. Entrapped microbial species within a restoration can survive within this nutrient-starved ecosystem by procuring glycoproteins and amino acid supplies from the pulpal fluids [20]. It has been suggested that the survival and growth of residual bacteria in such an adverse eco-system depend upon the ‘baseline’ pulpal health, remaining dentine thickness, the core composition of the residual microbiota, and the microbial load at the time of restoration [19, 21, 22]. For instance, in a recent seminal study, Marggraf and et al., [21] performed a kinetic assessment of *S. sobrinus* and *L. rahmnosus* beneath different restorative materials in stressed environments and noted that the survival kinetics of residual microbes were both strain-, and sealing- material-dependent.

The preceding, therefore, implies that a) microbes can survive and proliferate in stressed ecosystems even under sealed restorations, b) the composition, the load, and the species specificity of the residual microbiota determine their survival lifespan, c) the quality of the lesional floor and the dentine that entombs the restoration may be crucial for the microbial viability, and finally, d) the quality of the restorative material is an essential determinative factor. However, contemporary best practice does not, as yet, have any standardized microbiological-grounded approach in its caries management guidelines.

Given the variety of factors that affect the microbial survival within restorations, and the inability of the widely used restorative materials to destroy all the residual microbes in such locales, novel material formulations are imperative. The development and evaluation of this chemical armamentarium available in dentistry is necessary to tackle the issue of worldwide prevalence of rampant dentinal caries.

### Application of silver-compounds in dentistry

Elemental silver has been used as a biocide in a variety of biomedical applications for many a decade [23–26]. Indeed, silver in various formulations have been used since early 1900 to treat and arrest dental caries, and the metallic element incorporated in several dental products [27–29].

The exact mechanisms by which particulate silver kills bacteria and fungi are not precise, but several probable ways by which it may cause microbial destruction have been postulated [30, 31]. These include electrostatic bonding of silver ions to the anionic components of the microbial cell membranes, which appear to cause cell content leakage, abrogation of cell motility (in motile bacteria), and cell death. Second, Ag + ions appear to be toxic and thus poison the metabolic enzymes and block electron transport systems. Finally, they also seem to inactivate bacterial DNA and RNA via yet unknown pathways [32, 33].

Perhaps the earliest use of silver in dentistry was in the form of dental amalgam, which is a liquid mercury and metal alloy comprising mercury (50%), silver (22–32%), tin (14%), copper (8%) and other trace metals [34]. Another silver-containing compound, silver diamine fluoride (SDF), was introduced more than a decade ago to manage caries in primary and permanent teeth but was not particularly favored by the dental profession and gradually fell into disuse [35]. However, there has been a current resurgence of SDF usage, particularly for arresting and preventing dental caries progression in children [36, 37]. Interestingly, the Federal Drug Administration of the USA has approved the use of SDF for the dental and related purpose since 2014 [38].

Recent advances in manufacturing and biomaterial technology have led to the development of ‘second generation’ of silver products, and these include nano-molecules and nano-scaled silver nanoparticles (AgNPs). The particle size of nanomaterials ranges between 1 and 100 nm, and they appear to exhibit size-dependent physicochemical properties [39]. The bactericidal effect of AgNP is highest in the 1–10 nm size range, whence they exhibit highly reactive surface interactions [40].

The very small size and large contact area of nanoparticles have a remarkable effect on viable eukaryotic cells, influencing uptake efficiency, permeating cell membranes, and internalization, eventually leading to intracellular cytotoxicity and cell death, at high concentrations, [41] The high degree of cytotoxicity appears to be related to their nanoscale size, shape, surface area per unit mass, surface charge, and nonspecific oxidative damage [42, 43].

AgNP compounds currently used in dentistry include nanocomposites, adhesives, implant coatings, orthodontic material, and intracanal medicaments [29, 37, 38]. Due to the intense penetrative capacity and the microbicidal effects of AgNP, they appear to be good candidate chemicals for sanitization and disinfection of deep dentinal lesions, mainly to destroy the residual bacterial burden, after conservative caries removal (e.g., Atraumatic restorative treatment (ART).

Numerous in vitro and in vivo studies are available where silver compounds have been used in the management of human dental caries [27, 30, 36, 44–48]. However, to our knowledge, we are unaware of a systematic review that attempts to answer the question, “How effective is silver against cariogenic microbes in dentin biofilm?”

Hence, the primary objective of the present review was to systematically review the clinical application and antimicrobial potential of silver-containing formulation for caries arrest. Our secondary goal was to summarize the available in vitro evidence of microbicidal efficacy of silver-containing formulations against ‘cariogenic’ microbiota in dentine.

We believe that our findings reported here provide a state-of-the-art summary on the potency and the effectiveness of silver-compounds used in dentistry in eradicating cariogenic microbes in infected-dentine.

## Methods

### Data sources

Two investigators (LPS and KSF) performed an electronic search of English language manuscripts using Ovid MEDLINE, EBSCO host, Web of Science, and Cochrane Library databases. Published in vitro and in vivo studies were accessed between January 2009 and May 2019. A specific review question was formulated using the PICO framework as follows. Do silver-containing compounds (SDF/SDF + KI/AgNO_3_, nanosilver) (I) compared to distilled water/saline/Chlorhexidine (C) result in an efficient microbial reduction (O) in (P) dentin caries? The search keywords and combination of keywords were organized according to the PICO model, as illustrated in Table 1.

**Table 1:** Employed search terms and limits

Search strategy for systematic review of literature on the antimicrobial potential of silver-containing formulations (SDF, SDF/KI, Nano-silver, AgNO_**3**_)	
	**Search history**
**Search# 1**	KEY WORDS: (SDF and SDF/KI and AgNO_3_) (Jan 2009-May 2019)
**Cochrane Library**	Silver diamine fluoride AND antimicrobial AND AgNO_3_ AND dental caries AND silver nitrate OR caries arrest
**Pub med via OVID**	Silver diamine fluoride AND antimicrobial AND antibacterial AND dental caries OR dentine/dentin caries Silver diamine fluoride AND SDF AND SDF/KI antibacterial AND antimicrobial AND human dentine/dentin AND caries Silver diamine fluoride AND microbicidal/biocidal AND caries infected dentine AND caries affected dentine Silver diamine fluoride AND SDF/SDF/KI AND primary OR permanent teeth/dentition Silver diamine fluoride AND SDF/SDF/KI AND *in vitro*/*in vivo*/*ex vivo* OR clinical AND chemotherapeutic Silver diamine fluoride AND SDF/SDF/KI AND cariogenic AND microbes/bacteria AND caries arrest SDF AND SDF/KI AND dental caries AND dentine/dentin OR caries AND cavity/lesions
**EBSCO host and Web of Science**	Silver diamine fluoride AND microbicidal/biocidal AND caries infected dentine AND caries affected dentine Silver diamine fluoride AND SDF/SDF/KI AND primary AND permanent teeth/dentition Silver diamine fluoride AND SDF/SDF/KI AND *in vitro*/*in vivo*/*ex vivo* OR clinical AND chemotherapeutic Silver diamine fluoride AND SDF/SDF/KI OR cariogenic AND microbes/bacteria AND caries arrest
**Search# 2**	KEY WORDS: (nanosilver, AgNPs, AgNO_3_) (Jan 2009-May 2019)
**Pub med via OVID**	AgNPs AND silver nanoparticles AND caries infected/affected dentine AND dental caries Nanosilver AND AgNPs AND antimicrobial AND antibacterial AND dentine/dentin caries OR microbicidal/biocidal Nanosilver AND AgNPs AND silver nanoparticles AND human dentine/dentin AND caries Nanosilver AND AgNPs AND dental caries AND dentine/dentin OR caries AND cavity/lesions Nanosilver AND AgNPs AND silver nanoparticles AND AgNO_3_ AND primary AND permanent teeth/dentition Nanosilver AND AgNPs AND silver nanoparticles AND silver nitrate AND *in vitro/in vivo/ex vivo* OR clinical AND chemotherapeutic Nanosilver AND AgNPs AND silver nanoparticles AND cariogenic OR microbes/bacteria AND caries arrest
**EBSCO host and Web of Science**	Nanosilver AND AgNPs AND silver nanoparticles AND human dentine/dentin AND caries Nanosilver AND AgNPs AND dental caries AND dentine/dentin OR caries AND cavity/lesions Nanosilver AND AgNPs OR silver nanoparticles AND AgNO_3_ AND primary AND permanent teeth/dentition Nanosilver AND AgNPs AND silver nanoparticles OR silver nitrate AND *in vitro/in vivo/ex vivo* AND clinical AND chemotherapeutic AgNPs AND silver nanoparticles AND caries infected/affected dentine OR dental caries

### Study selection

Pre-determined inclusion criteria were:

**Clinical (in vivo*)*application:** 1) English language articles, 2) dentine/dentinal caries, 3) primary teeth, 4) permanent teeth, 5) ‘cariogenic’ microbes/bacteria, 6) antimicrobial effect, 7) pre-post microbial/bacterial reduction (caries arrest), 8) silver-based cavity disinfectants/formulations, and 9) asymptomatic dentine lesion.

**In vitro applications:** 1) English language articles, 2) in vitro pre-post microbial/bacterial reduction (caries arrest) on dentin-block- model, 3) human dentin/dentine (primary/permanent teeth), 4) ‘cariogenic’ microbes/bacteria, and 5) silver-based cavity disinfectants/formulations.

The exclusion criteria included: 1) review articles lacking related clinical data, 2) reports that do not allow extraction of data required to meet the set objectives, 3) enamel caries, 4) bovine dentin, 5) silver-based dental biomaterials, 6) studies with incomplete outcome details, 7) recruits (patient) on antibiotics, and 8) poster/conference presentation/abstracts. Grey literature and unpublished information through reading, were neither considered nor used; country or date enforced no limitations.

The identified research articles were compiled using bibliographic software, Endnote version 9. (Clarivate Analytics, USA).

### Electronic data search and analysis

**Fig.1:**
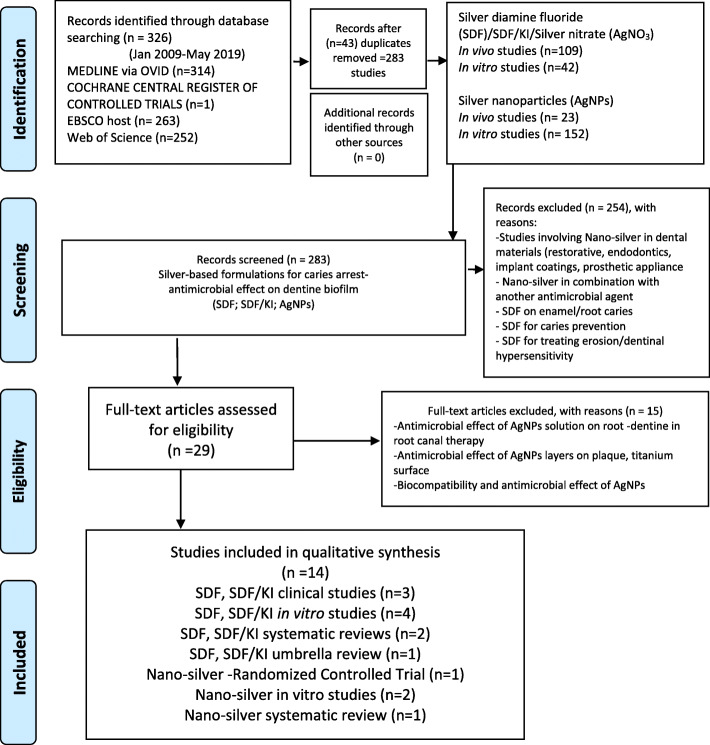
PRISMA flow-charts of the literature search and study selection

To ensure a systematic and comprehensive process, we followed the Preferred Reporting Items for Systematic Reviews and Meta-Analyses guidelines-PRISMA [49, 50]. The employed search strategy and results are summarized in Fig. 1.

The electronic data search and analysis were carried out in three stages. In stage one, the titles and abstracts of all identified relevant studies meeting predetermined inclusion criteria were screened. The full-text review was considered to have complete details of the data to make a precise decision. For the full-text review, the investigators (using spreadsheets) ensured that the eligibility criteria were met and that the outcomes meeting the study objectives were reported and retrievable. References were checked as a backward search of the included studies. To identify other studies that could be considered, a manual search of the listed references from the included articles was performed. In the third and final stage, the reviewer extracted and analyzed the data.

Using the Cochrane Collaboration risk of the bias assessment tool, the risk of bias was assessed to evaluate the methodological quality of the study [51]. This included an assessment of the method of randomization, allocation concealment, blinding of outcome assessment, selective reporting, and other sources of biases. Any discrepancies were discussed until mutual consensus was reached between the reviewers and the paper documented as low, unclear, or high risk.

### Data extraction

A total of 326 articles were identified after screening the different electronic databases, after which 43 duplicate publications were removed, leading to 283 studies that were initially reviewed. Of these, a total of 29 articles were identified and selected for full-text review as per the selection criteria for further analysis. The remaining publications were not considered as they were studies on enamel caries, caries prevention, erosion, or AgNP infused dental materials, that were unrelated to the present investigation. The employed search strategy and results are summarized in Fig. 1.

After reviewing the full text, in-depth detail of the characteristics of each study was recorded using the Cochrane pattern. This facilitated identifying the study design, setting and country, funding sources, specimen preparation, and test-microbes. Moreover, the type of intervention, comparator, evaluation time, assessment methods, sample size, statistical analysis, and study outcomes were comprehensively examined.

After an extensive review of the characteristics of the articles and drawing details from the tables evaluating the risk of bias of each trial, 14 studies were deemed suitable for the current systematic review (Table 2). Of 14-included studies, only three in vivo and five in vitro trials investigated the antimicrobial effectiveness of SDF, SDF/KI, AgNPs, AgNO_3_ in caries affected dentine of primary and permanent teeth. Summary of the characteristics of included studies and the reported results of antimicrobial efficacy of silver-compounds in these trials are summarized in Tables 3 and 4. Information detailing risk of bias assessment of each trial are given in Table 5.

**Table 2:** Reviewed Studies on SDF and AgNPs categorized as per the primary and secondary objectives of the review

		Silver diamine fluoride (SDF)	Nano silver particles (AgNPs)
***In vivo*studies** **Primary objective:** To systematically review the clinical application and antimicrobial potential of silver-containing formulation for caries arrest	**RCT**	Mitwalli et al., 2019 Milgrom et al., 2018 Karched et al., 2019	Santos et al., 2014
	**Systematic review- RCTs**	Urquhart et al., 2019 Trieu et al., 2019	(nil)
	**Umbrella review**	Seifo et al., 2019	(nil)
***In vitro*studies** **Secondary objective:** To summarize the available *in vitro* evidence of microbicidal efficacy of silver-containing formulations against ‘cariogenic’ microbiota in dentine	***In vitro*studies**	Mei et al., 2013(a) Chu et al., 2012 Mei et al., 2013(b) Hamama et al., 2015	Besinis et al., 2014 Schwass et al., 2018
	**Systematic review on*in vitro*studies**	(nil)	Noronha et al., 2017

**Table 3:** Major characteristics of the included studies

Study	Study design	Setting (deep dentinal lesion in primary/ permanent teeth)	Setting (human dentine blocks)	Inclusion/exclusion criteria	Test Microbes	Antimicrobial agents	No. of primary/ permanent teeth dentin blocks	Test & control group	Study Period
*In vivo* studies									
Karched et al., 2019	*In vivo* study	✓	**-**	✓	*Streptococcus mutans*	-38% Silver diamine fluoride -SDF/KI -2%CHX (+ve control)	25 permanent teeth	**-**	Evaluation after 48 hours of incubation
Milgrom et al., 2018	RCT- double blind placebo-controlled	✓	**-**	✓	Wide array of bacterial taxa (RNA sequencing)	-38% Silver diamine fluoride	6 primary teeth (2-caries lesion + 1-unaffected surface) (multi species)	**-**	Evaluation post SDF 14-21 days
Mitwalli et al., 2019	RCT	✓	**-**	✓	Wide array of bacterial taxa through DNA sequencing	-38% Silver diamine fluoride	20 permanent teeth (multi species)	**-**	Evaluation post SDF 4 weeks
*In vitro* studies									
Mei et al., 2013	*In vitro* study	-	✓	✓	*Streptococcus mutans* *Streptococcus sobrinus* *Lactobacillus acidophilus* *Lactobacillus rhamnosus* *Actinomyces naeslundii*	38% Silver diamine fluoride	Seventy- two dentin blocks (Biofilm model- mixed culture)	✓	Evaluation after 7, 14 and 21 days
Chu et al., 2012	*In vitro* study	-	✓	✓	*Streptococcus mutans* *Actinomyces naeslundii*	38% Silver diamine fluoride	Thirty-two dentin blocks (Biofilm model- mixed culture)	✓	Evaluation after 7 days
Mei et al., 2013(a)	*In vitro* study	**-**	✓	✓	*Streptococcus mutans* *Lactobacillus acidophilus*	38% Silver diamine fluoride	Thirty dentin blocks (Biofilm model- mixed culture)	✓	Evaluation after 7 days
Hamama et al., 2015	*In vitro* study	**-**	✓	✓	*Streptococcus mutans*	-Silver diamine fluoride/potassium iodide (SDF/KI) -Chlorhexidine (CHX) + SDF/KI	45 dentin blocks (Biofilm model-single species)	**X**	Evaluation after 5 minutes of dentine disc treatment
Besinis et al., 2014	*In vitro* study	**-**	✓	✓	*Streptococcus mutans*	-Nanosilver (AgNPs) -Silver nitrate (AgNO_3_) -Chlorhexidine	48, coronal dentin discs (Biofilm model-single species)	✓	Evaluation after 24-hours

✓=mentioned/present, in the included publication; X =not mentioned, in the included publication

**Table 4:** Results of the included studies

Study	Intervention and Comparator	Outcome Measure	Summary Intervention	Summary Comparator
Karched et al., 2019	-38% SDF ***vs.*** -SDF/KI ***vs.*** -2%CHX (+ve control)	***-Total viable count--Colony forming units***	**Median CFU counts/mg of dentine following SDF/KI treatment** 9x10^5^ to 1.6x 10^2^	**Median CFU counts/mg of dentine after CHX treatment** 1.6x10^3^ to 1.1x 10^2^
Milgrom et al., 2018	-38% SDF ***vs.*** -placebo blue tinted distilled water	***-RNA sequencing analysis to identify relative abundance of caries-associated microbes***	RNA sequencing analysis identified no consistent changes in the relative abundance of cariogenic microbes	
Mitwalli et al., 2019	-38% Silver diamine fluoride	***-16S rDNA sequencing to determine bacterial profiles***	**Decrease in count of acidogenic bacterial species post-SDF treatment** *Actinomyces sp. HOT448* *Propionibacterium acidifaciens* *Scardovia inopinata* *Propionibacterium sp. HOT194* *Treponema denticola* *Bifidobacterium dentium* *Propionibacterium genus probe* *Parascardovia denticolens* *Scardovia genus probe 4* *Actinomyces israelii*	
Mei et al., 2013	-38% SDF ***vs.*** -Distilled water	***-Microbial Kinetics-Colony forming units*** ***-Viability of microbes-Confocal microscopy***	**Day 21** (total bacteria) 5.72±0.19 **Day 21*Dead: live ratio*** *0.92±0.36*	**Day 21** (total bacteria) 12.20±0.84 **Day 21*Dead: live ratio*** *0.004±0.001*
Chu et al., 2012	-38% SDF ***vs.*** -Distilled water	***-Microbial Kinetics-Colony forming units*** ***-Viability of microbes-Confocal microscopy***	*S. mutans****(****Log CFU=0)* *A. naeslundii (Log CFU=0)* *S. mutans****Dead: live ratio*** *5.61±2.42* *A. naeslundii****Dead: live ratio*** *16.01±10.83*	*S. mutans (Log CFU=6.03±0.18)* *A. naeslundii (Log CFU=7.00±0.24)* *S. mutans****Dead: live ratio*** *0.025±0.01* *A. naeslundii****Dead: live ratio*** *0.23±0.09*
Mei et al., 2013(a)	-38% SDF ***vs.*** -Distilled water	***-Microbial Kinetics-Colony forming units*** ***-Viability of microbes-Confocal microscopy***	*S. mutans****(****Log CFU=4.02±0.35)* *L.acidophilus (Log CFU=1.80±0.28)* *S.mutans and L. acidophilus* ***Dead: live ratio*** *6.74±5.29*	*S. mutans (Log CFU=6.03±0.18)* *L.acidophilus (Log CFU=7.00±0.24)* *S.mutans and L. acidophilus* ***Dead: live ratio*** *0.02±0.002*
Hamama et al., 2015	-SDF/KI ***vs.*** -Chlorhexidine	***-Mean percentages of dead and live bacteria****after 5 minutes of surface treatment*	**SDF/KI group** (56.1 ±13.0%) **SDF/KI following Carisolv** (27.7 ±13.5%) **SDF/KI following Papacarie gel** (36.9 ±4.4%) **SDF/KI group** (56.1 ±13.0%)	**Carisolv** (0.4± 0.7%) **Papacarie** (19.5± 6.6%) **CHX** (19± 10.8%) **CHX vs. Papacarie gel** (19.5± 6.6%)
Besinis et al., 2014	-Nanosilver (AgNPs) ***vs.*** -Silver nitrate (AgNO_3_) ***vs.*** -Chlorhexidine	***- Measuring turbidity,*** ***-Proportion of live and dead cells and*** ***-lactate production***	All three bioassays demonstrated both silver nanoparticles and silver nitrate dentine coatings highly bactericidal (>99.5%) against *S. mutans*	Chlorhexidine coating on dentine disc showed no antibacterial effect against *S. mutans*

**Table 5:** Risk of Bias of the included studies

Study	Selection bias Baseline characteristics similarity\ appropriate control selection	Selection bias Allocation concealment	Selection bias Randomization	Performance bias Blinding of Researchers	Detection bias Blinding of outcome assessors	Reporting bias Selective outcome reporting	Confounding bias Account for confounding variable
*In vivo* studies							
Karched et al., 2019	**+**	**?**	**+**	**?**	**?**	**?**	**+**
Milgrom et al., 2018	**+**	**?**	**+**	**?**	**?**	**?**	**-**
Mitwalli et al., 2019	**+**	**?**	**+**	**-**	**?**	**?**	**-**
*In vitro* studies							
Mei et al., 2013	**+**	**?**	**+**	**?**	**+**	**+**	**?**
Chu et al., 2012	**+**	**?**	**+**	**?**	**?**	**+**	**?**
Mei et al., 2013(a)	**+**	**?**	**+**	**?**	**?**	**+**	**?**
Hamama et al., 2015	**+**	**?**	**+**	**?**	**+**	**+**	**?**
Besinis et al., 2014	**+**	**?**	**+**	**?**	**?**	**+**	**?**

Risk of bias legends: + (Low risk); - (High risk); ? (Un-clear risk)

## Results

The primary objective of the present review was to systematically review the clinical application of silver-based formulations as an efficient biocide against cariogenic microbes of dentin. Therefore, the results are discussed below in two stages. First, as in vivo studies with a summary of presented information with the reviewers’ assessment and critical appraisal/ comments and then as in vitro studies featuring an overview of the existing data in the literature, together with the reviewers’ appraisal.

### Primary objective: clinical application and antimicrobial potential of silver-containing formulation (SDF and AgNPs) for caries arrest

#### Silver diamine fluoride (SDF)

Two retrieved clinical studies investigated the effect of SDF treatment on primary and permanent teeth on an array of bacterial taxa.

##### Silver (Ag+) compounds 38%SDF- in the management of dentine caries in primary teeth

Milgrom and colleagues’ (2018) investigated the microbicidal effects of SDF against a wide-ranging microbial species isolated from inactive, cavitated-caries lesions [52]. They performed a randomized, double-blind, placebo-controlled trial with parallel groups of three 66 preschool children. Plaque samples from an unaffected, healthy tooth surface, and two caries active lesions were collected before, and after 2 to 3 weeks (14–21 days apart) of 38%SDF application. To enable the measurement of viable microbes, they used an RNA sequencing method.

Intriguingly, Milgrom et al. observed no relative reduction in caries-associated microbial-load and noted a marginally increased and insignificant burden of non-cariogenic microbes and minimal microbial diversity, in the SDF-treated group. They concluded that silver-containing compounds (SDF) showed a non-selective microbicidal effect on all microbes present inactive caries lesions rather than a selective microbicidal effect. Clinically, they observed an apparent caries arrest in over 70% of the SDF treated lesions, confirmed through both visual and tactile assessments using probes.

##### Silver (Ag+) compounds 38% SDF)- in the management of cervical and root-dentine caries in permanent teeth

Very recently, Mitwalli et al. [53], employing a 16S rDNA sequencing method, assessed the biocidal effects of 38% SDF on a broad-range of microbiota present in cervical and root-dentin caries-lesions. Twenty-healthy patients with at least one asymptomatic tooth with caries severity ICDAS code5/6 (distinct cavity with visible dentine extending half/more than half of tooth surface) were randomly selected and treated with SDF. Plaque samples were collected before and 1-month post-SDF treatment from the clinically confirmed lesions.

Similar to the observations of Milgrom et al. [52], they noted that SDF intervention had no significant effect on the bacterial composition of the dentinal lesions, one-month post-application. However, they observed a trend towards a relative reduction in the abundance of some acidogenic taxa, post-treatment. These taxa included *Scardovia* spp., *Actinomyces* spp., and propionibacterium implicated in caries progression.

##### Silver (Ag+) compounds SDF & SDF/KI- in the management of dentine caries in permanent teeth

An in vivo study by Karched et al. [54] compared the microbicidal efficacy of two silver-based compounds SDF and the latter together with potassium iodide (SDF + KI), on cariogenic microbes in coronal dentine-caries lesions; chlorhexidine (2%) and sterile saline were used as positive and negative controls, respectively. The biocidal efficacy of these compounds was assessed in a single tooth, each in five adult recruits. They selected asymptomatic teeth with clinical evidence of caries (in the inner one-half of the dentin thickness) that was confirmed by radiographs and pulp vitality tests. Traditional phenotypic microbiological assessment of dentine samples pre- and post- SDF and SDF/KI applications, exhibited virtually complete elimination of *mutans*-streptococci after 48-h post-application in four of the five patients. Furthermore, a significant reduction in the total viable counts by over 95% anaerobes was also reported after the SDF + KI application.

##### Methodological quality assessment

In general, we found the following methodological concerns in the aforementioned clinical studies:

*Assessment bias*: In interventional studies, for the establishment of a true treatment-effect, confounding variables must be suitable adjusted [55]. Milgrom et al. and Mitwalli et al. [52, 53] showed no significant differences in the microbial profile and diversity after 2 and 4 weeks- post-SDF application, respectively. Their studies are, to some extent, weak as measurable confounders such as diet, salivary flow, and oral hygiene status of the tested patient cohorts were not recorded. Besides, possible bacterial re-colonization in an open-lesion during post-treatment span could also have impacted their results. As these unevaluated confounders may obfuscate the treatment effects their findings may be open to criticism.

*Outcome measurement:* Both Milgrom et al. and Mitwalli et al. [52, 53] reported caries-arrest in the post-SDF-treated caries lesions, by evaluating the tactile hardness or the lesion texture of the cavities. Surprisingly, the post-intervention caries-arrest reported by Milgrom et al. [52] did not compare the relative microbial profiles of the treated and untreated lesions by available molecular microbiological analytical methods. Clearly, for a valid assessment of caries progression /arrest, both qualitative (visual & tactile) and quantitative (microbial reduction) assessments should be considered. The very high subjectivity and the low sensitivity of the tactile examination results make the outcomes of Milgrom et al. and Mitwalli et al. [52, 53] study somewhat questionable.

*Blinding*: Allocation process in Karched et al. [54] and Milgrom et al. [52] studies were stated as double-blinded. However, the blinding of outcome assessors is unclear in both these studies. Additionally, the clinical trial of Mitwalli et [53]. also lacks blinding, as no placebo treatment was given to these patients. Thus the blind status of all three of the above studies is questionable.

*Sample size*: Small sample size in clinical trials have limitations, as this can significantly affect the conclusions of the reported trials and thus affect the inferences or extrapolations drawn [56]. The difference between the sample sizes of the three reviewed clinical trials was extreme, varying from three to 20 samples. Therefore, the results need to be cautiously interpreted and need further validation.

*Duration*: In the three included clinical studies, the period between SDF-application and post-treatment sample collection varied from a few minutes to 2-and 4-weeks, which clearly can have a significant impact on the outcomes.

Additionally, the included clinical trials have some limitations. Though, randomization was reported in these studies but without detailed explanation in their methodology. Moreover, allocation concealment was unclear in all the included RCTs.

##### Systematic reviews on SDF in randomized clinical trials-(RCT)

There are three noteworthy reviews on RCTs published in 2019 summarising the effectiveness of SDF under a variety of scenarios (Table 2). The first, a systematic review by Urquhart et al. [57], summarizes the available evidence on the effectiveness of various interventions for non-restorative, management of carious lesions. They reviewed 44 studies that included parallel or split-mouth randomized controlled trials, with follow-up periods of varying lengths. They compared the relative efficacy of a total of 22 different interventions in a cumulative sample of over 7000 participants.

The randomized controlled trials (RCTs) included interventions for arresting, either non-cavitated or cavitated caries lesions in both the primary and permanent dentition. Interventions included a broad spectrum of modalities such as silver compounds (SDF and AgNO_3_), varying fluoride and calcium-containing formulations, polyols, chlorhexidine, casein phosphopeptide–ACP (CPP-ACP), nano-hydroxyapatite, prebiotics, lasers, resin infiltration, sealants, sodium bicarbonate, and carbamide peroxide. They concluded that 38% SDF solution application was the most effective compared to other examined interventions, as determined by assessing the clinical texture of the treated lesions.

The second, recent systematic review and meta-analysis by Trieu et al. [58] compared caries arresting the potential of SDF and a conventional sodium fluoride formulation. A total of six -reviewed studies (including 2-RCTs) including over 700 pre-school children as recruits were studied. Caries arrest was evaluated by visual means, and tactile sensitivity of the lesion hardness by manual assessment, after SDF application.

Finally, Seifo et al. [59], in an umbrella review, have comprehensively evaluated and summarized systematic reviews on silver diamine fluoride potential in preventing and arresting caries trials published between 1970 and 2018. Of the 11 included systematic reviews, four focused on SDF efficacy in arresting root caries, and seven, coronal caries in children. They concluded that the review data consistently support the superior effectiveness of SDF in caries arrest. They significantly outpaced other formulations competing for therapeutic approaches, including fluoride varnish application, and atraumatic restorative treatment.

*Outcome reporting bias*: Virtually all the reviewed evidence on the effectiveness of the SDF application was based on conventional, subjective visual and tactile clinical examination of caries lesion hardness, and this appears to be the significant universal bias in the previously reported studies.

Given that dental caries is a sugar-biofilm dependant disease leading to alterations in the tooth tissue hardness [60], at present, there appears to be a no-surrogate indicator for measuring this element apart from the somewhat subjective, tactile clinical examination, to evaluate the disease outcome. Clearly, the sensitivity, precision, and reliability of the latter technique are questionable. Hence a more valid quantitative indicator of caries arrests, such as a reduction in the burden and nature of potentially cariogenic microbiota, pre- and post-intervention, would be a more sensitive and reliable indicator. The arrival of next-generation sequencing (NGS) technology and the novel miniaturization methods [61, 62] should go some way in addressing this crucial issue and should be adopted by the research community.

#### Silver nanoparticles (AgNPs)

Management of dental caries with nano-scaled silver particle (AgNPs) formulations is perhaps the most recent clinical advance in cariology. There are a but, very few studies on this subject, and we discuss below a clinical trial that investigated the impact of chitosan-capped silver nanoparticle solution on caries arrest in the primary teeth of pre-school children.

##### Silver nanoparticle (AgNP) compounds in the management of dentine caries in primary teeth-a randomised controlled trial

Santos et al. [63] conducted a prospective, randomized, double-blinded, controlled clinical trial on 60 children, mean-aged-6-years-old. Single examiner using the ICDAS criteria for caries lesion activity, and the diagnosis was used to evaluate the lesions. Later, the same examiner applied nano-silver fluoride (NSF; 2 drops/tooth; equivalent to a dose of 10 mg of the solution), using the micro brush on 63-preselected teeth of children in the test-group. In the control group, they tested (1drop/tooth) of NSF, on 67-selected teeth. Another calibrated examiner followed the test and control groups. They reported ‘caries arrest’ of up to 81, 72.7, and 66.7% caries-arrest at 7-days, 5 and 12 months- post-treatment follow up, respectively. Nevertheless, again, their instrument/unit of evaluation of caries-lesion hardness pre-and post-treatment was the tactile measurement.

##### Methodological quality assessment

Methodological quality assessment caveats of the Santos et al. [63] study include, i) Missing data on measurable confounding factors such as diet and oral hygiene status, ii) unclear description of baseline caries lesion severity of the treated and control groups, and finally as reported above iii) evaluating clinical caries arrest by subjective, tactile-hardness assessment.

Due to these several methodological biases in the single RCT on the efficacy of silver nanoparticles on caries arrest, it could be concluded that the currently available evidence is of poor quality.

### Secondary objective: in vitro application of silver-containing compounds (SDF and AgNP) in dentistry. (Table 2)

#### Silver diamine fluoride

##### In vitro application of SDF on dentine caries lesions

We reviewed four in vitro trials using ex vivo dentine block models (Table 2), where test and the controls were evaluated after allowing the bacteria to grow and mature into a biofilm, mimicking clinical carious dentine [64–67]. Employed methodology and outcome measures between these studies were similar, and significant differences were documented between primary and permanent teeth regarding microbes, dentine thickness, morphology, and structure. The data from the in vitro models indicate that SDF can efficaciously reduce residual cariogenic bacteria in the dentine of both primary and permanent dentitions. Additionally, these studies clearly demonstrate the antimicrobial efficacy of 38% SDF against specific dentinal microbes [64–67].

Nonetheless, there are limitations of the four investigations [64–67]. First, researchers assessed standardized, artificially produced lesions with well-defined dentine thickness. Second, although the plaque microbial spectrum is vast in natural lesions, the tests evaluated the SDF antimicrobial effects only on two to five caries associated microbial strains, with outcome measure being the number of colony-forming units (CFU). Three studied, however, reported the efficacy of SDF through alternative approaches such as imaging, employing live-dead staining technology through scanning electron, and confocal microscopy, in addition to CFU counts [64–66]. Plainly, such in vitro methods can evaluate only the cultivable bacteria and not the total microbiome, which includes both cultivable and uncultivable organisms.

In general, the effect of the frequency and duration of the SDF application could not be evaluated due to the short period of evaluation used in these in vitro studies. Further, due to the lack of external validity and generalizability to real clinical situations, the results should be interpreted with caution.

#### Silver nanoparticle (AgNP)

##### In vitro *application of silver nanoparticle (AgNP) formulation -as tooth disinfectant for treating dental caries* (Table 2)

Besinis and team [68] tested in vitro biocidal efficacy of silver nanoparticle (56.8 nm) and silver nitrate (52.8 nm) against mono-species cariogen, *S. mutans* on human dentine discs (*n* = 48). They compared the antimicrobial activity of silver compounds with chlorhexidine. Microbial growth and cell viability were quantitatively evaluated by measuring the turbidity, determination of live/dead cells, and lactate production. Both silver-containing solutions exhibited an equally powerful antimicrobial effect (> 99%) while impeding microbial adhesion on the dentine surfaces. Chlorhexidine did not offer any additional protection compared to the uncoated dentine control, while silver nitrate caused dentine discoloration.

Another recently published in vitro study by Schwass et al. [69] tested AgNPs formulation as a targeted application for disinfecting carious dentine. Colloidal suspensions of 6.7–9.2 nm stabilized AgNPs were tested against planktonic cultures as well as monoculture biofilms of selective microbes (*Streptococcus mutans, S. mitis, S gordonii, Pseudomonas aeruginosa, Enterococcus fecalis*). Biocidal effects were compared with SDF, and 70% isopropanol used as controls. Compared to 38% SDF, the preparation of AgNPs used low concentrations of silver (38.4 μg/ml vs. 320,000 μg/ml). Antimicrobial activity was tested using agar diffusion, biofilm sensitivity, and crystal violet assays. Schwass et al. [69] noted that AgNP suspension inhibits in vitro monoculture biofilm formation of the evaluated *Streptococcus* spp. as well as *E. fecalis.*

The outcomes and the clinical values of these in vitro studies are limited as definitive conclusions on the relative therapeutic efficacy of nano-silver can be drawn only for a limited number of cariogenic microbes of coronal dentine. However, the data are valuable as a basis for further studies with other microbiota implicated in dentinal caries.

##### Systematic review of AgNPs including in vitro trials*:* (Table 2)

In a comprehensive study, Noronha and colleagues (2017) reviewed 155 peer-reviewed articles, published between 2012 and 2017, on the applications of AgNPs in dentistry [70]. The authors included 7-in vitro trials which tested the biocidal activities of nanosilver in pre-formulations, against known cariogenic microbes.

The reviewers concluded that the formulations containing AgNPs demonstrated size-dependant antimicrobial effects. In the reviewed studies [47, 48, 71], nanoscaled- silver, of size ranged between 5 and 20 nm, showed a promising biocidal effect on the cariogens belonging to *Streptococcus* species. The reviewers also mentioned the limited information available on the antimicrobial effect of nanosilver on the biofilm phase, as opposed to the planktonic (suspended) phase of cariogenic microbes. However, they concluded that nanosilver formulations have a promising potential in managing caries, although data on the safety of nanosilver in the oral cavity is not extensive. Hence, further in vitro as well as animal studies elucidating the efficacy and safety of nanosilver followed by clinical trials are warranted to unequivocally ascertain the status of AgNPs as a promising anti-caries formulation worthy of routine clinical use. Finally, with increasing popularity and advances in nanoparticle technology, as well as the currently extant data, reviewed above, it is likely that in the fullness of time, the popularity of nano-chemicals will increase as the main stream method of caries management in clinical practice.

Last of all, the included clinical trials in the present review are diverse (clinical heterogeneity). Milgrom et al., [52] and Mitwalli and colleagues [53] studies reported post-treatment SDF-effect on a wide array of bacterial taxa employing RNA (meta-transcriptomic analysis) and DNA (metagenomic sequencing methods), respectively. Whilst, Karched and team reported therapeutic efficacy (microbial load reduction) of 38% SDF against selective cariogenic microbes of coronal dentine. Therefore, to combine all included clinical trials in a meta-analysis possibly could have obscure the genuine differences in effects of the treatment. This limits the possibility for meta-analysis, intended to obtain a meaningful assumption from the data. Likewise, it was difficult to meta-analyze the pre-post differences in mean CFU/ml after 38% SDF application on carious dentine, due to varying post-treatment evaluation times ranging between 5-min to 7/14 or 21-days of in vitro trials.

## Conclusions

The present review on the application of silver- formulations as anti-caries agents, has identified following concluding notes:

1. The reviewed in vitro studies in the current analysis showed good evidence of microbicidal efficacy of silver containing-SDF formulations on selective cariogenic microbes in human dentine models.
2. There is conflicting evidence of antimicrobial efficacy of silver diamine fluoride in clinical settings. The clinical trials of two and four-weeks-post SDF treatment reviewed here do not provide strong evidence on a high degree of effectiveness of the material on a wide array of cariogenic microbes. Though, there is some evidence to show that SDF is possibly an effective microbicide against specific cariogens belonging to the *Streptococcus* genus. A significant biocidal effect, in particular, on anaerobes was also noted with a combination of SDF/KI treatment.
3. There is good evidence of the in vitro application of silver nanoparticles as a useful antimicrobial against *S. mutans* adhesion, growth, and subsequent biofilm formation in dentine lesions.
4. There is insufficient evidence in the application of AgNP-fluoride as an effective microbicidal in clinical settings.

In conclusion, the present review of the available in vitro studies has shown the promising antimicrobial potential of silver-based formulations (SDF and nanosilver) against the predominant cariogenic flora of dentine lesions. Post-treatment data of either the bactericidal and bacteriostatic effects of SDF or nano-silver are sparse. Furthermore, the current understanding of the specific size, concentration, antimicrobial mechanisms, and toxicological aspects of nano-silver is insufficient. To justify the routine use of silver-based compounds for anti-cariogens, there is a need to generate the highest level of evidence by carefully standardizing the methods of basic experimentation protocols, outcome assessment indicators, and analysis.

Evidently, then, long term clinical trials are warranted to obtain robust evidence to base conclusive recommendations. Such trials should address the gaps in our knowledge on the optimum dosage and therapeutic regimens necessary for the routine use of silver containing microbicides against polymicrobial consortia of caries-affected dentine.

## Supplementary information

**Additional file 1.**

## Data Availability

All required data and supplementary materials will be available for on request.

## Abbreviations

SDF: Siver diamine fluoride
AgNPs: Silver nanoparticles
SDF/KI: Silver diamine fluoride/Potassium Iodide
CFU/ml: Colony forming unit/milliliter
ACP (CPP-ACP): Amorphous Calcium Phosphate (Casein phosphopeptide- amorphous calcium phosphate)
AgNO_3_: Silver nitrate
