# Supplementary material for: Clinical efficacy and the antimicrobial potential of silver formulations in arresting dental caries: a systematic review
Source: BMC Oral Health. 2020 Jun 3;20:160. doi: 10.1186/s12903-020-01133-3 (PMC7268710; doi:10.1186/s12903-020-01133-3)
Supplement: Supplementary file 1 — Additional file 1. [file 12903_2020_1133_MOESM1_ESM.docx]

**CHARACTERISTICS OF STUDIES**

CHARACTERISTICS OF INCLUDED STUDIES

**Mei 2013(a)**

| **Title** | **Antibacterial effects of silver diamine fluoride on multi-species cariogenic biofilms on caries** |
| --- | --- |
| **Material and Methods** | **Design:** *in vitro study*  **Setting:** *in vitro study* using human dentin blocks in a computer controlled artificial mouth  **Country:** Hong Kong, China**.**  **Funding sources:** General Research Fund (HKU765111M) of Research Grant Council, Hong Kong |
| **Materials** | **Artificial mouth preparation:**   - All dentin blocks incubated in the computer controlled artificial mouth at 37^0^C for the growth of biofilm in a controlled environment - A humidified mixture of 5% CO_2_ and 95% Nitrogen was supplied continuously at 60ml/min. - To mimic salivary flow, simulated oral fluid was supplied at 2.5ml/hr. - 5% sucrose solution was supplied for 6 mins with a flow rate of 15ml/hr for every eight hours. - Artificial oral condition was monitored by a computer program (LabVIEW software Version 2.2). - **Inclusion criteria**: extracted sound third molars stored - **Exclusion criteria:** not reported   **Specimen preparation:** Seventy-two dentin blocks (2x2x4mm^3^) prepared from sound human third molars   - Three sets of 24 dentin blocks each were prepared. - Of a set of 24 dentin blocks, 12 blocks embedded in acrylic resin disc designed to fit inside computer controlled artificial mouth, were sterilized with ethylene oxide for 16 hours in this in vitro study - Five equal aliquots of 10^7^CFU/ml of five organisms were mixed and inoculated on dentin blocks and incubated an-aerobically at 37^0^C for 3 days in brain-heart infusion broth to create carious lesion of 70µm in depth - Dentin blocks were divided into two equal groups (test and control) - Of twenty-four blocks:   **Test group: 12** Dentin blocks assigned in the test group, were treated with 0.22mg of 38% SDF (8.8µg fluoride) topically  **Control group: 12** Dentin blocks assigned in the control group were treated with sterile distilled water topically  **Evaluation after 7 days:**   - First set of 24 blocks were evaluated after 7 day-period after allowing the bacteria to grow and mature into biofilm   **Evaluation after 14 days:**   - Another 24 dentin blocks were prepared and assessed similarly after 14 days of bacterial growth and maturation into biofilm   **Evaluation after 21 days:**   - Remaining 24 dentin blocks were prepared and assessed similarly after 21 days period. |
| **Methods** | **Biofilm Assessment**  **CFU counting:**   - For specific identification of individual species, selective media plates were used   Mitis Salivarius agar plate for Mutans Streptococci identification  Rogosa agar plate for Lactocacilli identification  Actinomyces agar plate for Actinomyces identification   - Isolated bacterial colonies were confirmed by Gram stain and Catalase test   **Surface topography of the biofilm:** to study viability and distribution of the biofilm   - To denote the ratio of dead-to-live bacteria, the bacteria on the dentin surfaces were labeled in situ using two fluorescent probes: propodium iodide and SYTO-9 dye - Ten cellular images of the middle layer of each biofilm obtained using Confocal laser scanning microscopy (CLSM) and analyzed using an image analysis software (Image J)   **Dentin block Assessment**  **Evaluation-hardness of carious lesions:**   - Five sets of Knoop hardness number (KHN) measurements were made on each block, determined at 25 μm below the surface of the dentin blocks in increments of 25 μm on both experimental and internal control sites - Relative microhardness was calculated from the measured KHN on the carious dentin, divided by the KHN on sound dentin   **Evalauation-mineral content of the carious lesions:**   - Elemental analysis of calcium (Ca), phosphorus (P), and the Ca/P ratio was assessed by energy dispersive spectroscopy (EDS) in a region close to the microhardness indentations   **Evaluation- potential changes in the organic structure of the carious lesions:**   - Calculations were made from the spectrally derived matrix-to-mineral ratio (the ratio of the integrated area of protein amide I absorbance from 1585 to 1720 cm^-1^ to that of phosphate [HPO_4_^2-^] absorbance from 900 to 1200 cm^-1^) |
| **Appropriate Statistical methods** | **Sample size determination:**   - Not reported   **Statistical Analysis:**   - Shapiro-Wilk test for a normal distribution - Parametric t test was used to compare log CFU, relative microhardness, weight percentage of Ca and P, Ca/P ratio, and log [amide I: HPO_4_^2-^] ratio between the test and control groups |
| **Test Organisms** | **Test microbes:**   - Streptococcus mutans ATCC (American Type Culture Collection) 35668 - Streptococcus sobrinus*ATCC 33478* - Lactobacillus acidophilus*ATCC 9224* - Lactobacillus rhamnosus*ATCC 10863* - Actinomyces naeslundii*ATCC 12014* |
| **Outcomes** | **Outcomes:**   - Reduction in total bacterial count in caries affected dentin - Reduction in viable lactobacilli count in caries affected dentin   **Time points:** Assessment after 24 hours of incubation  **Diagnostic criteria for caries free dentin:**   - Not reported - Visual and tactile assessment using dental probe |
| **Notes** | - *In vitro* study, using human third molar dentine blocks, placed in a computer controlled artificial mouth - Investigated SDF effect on multi species **(*S. mutans, S. sobrinus, L. acidophilus, L. rhamnosus, and A. naeslundii*)** biofilm - Reported CFU count of multi species biofilm after treatment with SDF solution - Assessed viability and physiological activity of bioﬁlms using Confocal laser scanning microscopy (CLSM) - Assessed micro hardness and changes in mineral content of the dentine carious lesion |

**Mei 2013(a)**

| **Title:** **Antibacterial effects of silver diamine fluoride on multi-species cariogenic biofilms on caries** | | |
| --- | --- | --- |
|  | **Risk of bias** | **Supporting reference and comments** |
| **Selection bias** |  |  |
| Baseline characteristics similarity/appropriate control selection | Low risk | QUOTE: "All of the blocks were then incubated in the artificial mouth, which provided a controlled environment for the growth of the biofilms".  "A humidified gas mixture of 5% carbon dioxide and 95% nitrogen was supplied continuously at 60 ml/min. The temperature inside the artificial mouth was maintained at 37°C. Simulated oral fluid (defined medium mucin) was continuously supplied at 2.5 mL/hr to mimic salivary flow. Sucrose solution at 5% was supplied for 6 minutes every 8 hours with a flow rate of 15 mL/hr. The conditions were monitored by a computer program (LabVIEW® software Version2.2)"  COMMENT: Complete detail of controlled environment for the growth of the biofilms using artificial mouth is given |
| Allocation concealment | Unclear risk | Not reported  COMMENT: Probably done |
| Randomization | Low risk | QUOTE: Half of the blocks were assigned as a test group, and the other half were used as a control.  COMMENT: Probably done |
| **Performance bias** |  |  |
| Blinding of Researchers | Unclear risk | Not reported  COMMENT: Probably done |
| **Detection bias** |  |  |
| Blinding of outcome assessors | Low risk | QUOTE: The CFU counting results were re-checked by a technician who do not know the experiment design  COMMENT: For biofilm assessment (re-checking of CFU count) by a technician not aware of the study is specified |
| **Reporting bias** |  |  |
| Selective outcome reporting | Low risk | COMMENT: All expected outcomes reported |
| **Confounding bias** |  |  |
| Account for confounding variable | Unclear risk | Not reported  COMMENT: Probably done |

**Chu 2012**

| **Title** | **Effects of silver diamine fluoride on dentine carious lesions induced by *Streptococcus nutans* and *Actinomyces naeslundii*** |
| --- | --- |
| **Material and Methods** | **Design:** *in vitro study*  **Setting:** *in vitro study* using human dentin blocks  **Country:** Hong Kong, China**.**  **Funding sources:** Study was supported by Grant No. 00802159003 of Seed Fund for Basic Research, University of Hong Kong |
| **Materials** | **Sample size calculation:**  Assuming the mean lesion depth before and after the 7-day bacterial demineralization challenge were 100 to 150µm and the common standard deviation was 35 µm. The sample size was at least eight in each group with power at 0.80 and ᾱ=0.05.  **Specimen preparation:** Thirty-two dentin blocks (2x2x4mm^3^) prepared from sound human third molars   - **Inclusion criteria**: Extracted sound human third molars stored in 1% sodium azide at 4^0^C - **Exclusion criteria:** Observation of dentin blocks under stereomicroscope (x10 magnification) exhibiting cracks, hypoplasia or white spot lesion were excluded - Half of the surface of each block was coated with two layers of an acid-resistant nail varnish as internal control - To facilitate and speed up the development of carious lesions by cariogenic bacteria, each dentin block was incubated in an acidified buffer containing 50mM acetic acid, 2.2 mM KH_2_PO_4_ and 2.2mM CaCl_2_ at pH 4.4 for 96hrs for 23^0^C - The blocks were then sterilized with ethylene oxide for 16 hours   **Dual-species cariogenic biofilm (S. mutans and *A. naeslundii*)**   - Cultured on blood agar plates at 37^o^C for 2 days an aerobically - A single colony from each plate was picked to prepare 24-hour broth cultures in basal medium supplemented with 5% glucose BMG medium at 37^o^C under anaerobic conditions - Bacterial suspensions were then prepared in BMG to a cell density of McFarland 4(10^9^ cells/ml)   **Inoculation of dentin discs with S. mutans and *A. naeslundii***   - A 300µL aliquot (4x10^9^ cells/ml) of each bacteria culture was mixed and inoculated on each dentine block, placed in a well of 24-well plate BMG, in an anaerobic chamber with 95% nitrogen and 5% carbon dioxide at 37^o^C for 7 days. - The medium was refreshed daily, without disturbing the specimen surface - After 7 days, eight blocks in each bacteria group underwent topical application of 38% SDF on exposed surfaces with a microbrush - After treatment all the dentine blocks were placed in incubator shaker at 75rpm inside the anaerobic chamber for 7 days at 37^o^C   **Evaluation after 7 days:**   - Test and control blocks were evaluated after 7 day-period after allowing the bacteria to grow and mature into biofilm |
| **Methods** | **Biofilm Assessment**  **CFU counting:**   - After serial dilution growth kinetics of the mono-species biofilm for 7 days was assessed by determining the bacterial counts in colony forming unit   **Scanning electron microscopy (SEM) -topographical features of the biofilms:**   - Biofilm samples were rinsed, washed, dehydrated and dried in a desiccator and sputter coated with gold - To examine the topographical features of the biofilms, scanning electron microscopy (SEM) was used at 12kV in high-vacuum mode   **Surface topography of the biofilm *and Confocal laser scanning microscopy*:** to study viability and distribution of the biofilm   - To denote the ratio of dead-to-live bacteria, the bacteria on the dentin surfaces were labeled in situ using two fluorescent probes: propodium iodide and SYTO-9 dye - Four cellular images of each biofilm obtained using ***Confocal laser scanning microscopy (CLSM)*** and analyzed using an image analysis software ***(Image J)***   **Dentin block Assessment**  **Evaluation-hardness of carious lesions:**   - Five sets of Knoop hardness number (KHN) measurements were made on each block, determined at 25 μm below the surface of the dentin blocks in increments of 25 μm on both experimental and internal control sites - Relative microhardness was calculated from the measured KHN on the carious dentin, divided by the KHN on sound dentin   **Evalauation-mineral content of the carious lesions:**   - Elemental analysis of calcium (Ca), phosphorus (P), and the Ca/P ratio was assessed by energy dispersive spectroscopy (EDS) in a region close to the microhardness indentations   **Evaluation- potential changes in the organic structure of the carious lesions:**   - Calculations were made from the spectrally derived matrix-to-mineral ratio (the ratio of the integrated area of protein amide I absorbance from 1585 to 1720 cm^-1^ to that of phosphate [HPO_4_^2-^] absorbance from 900 to 1200 cm^-1^) - The log value of [amide I: HPO_4_^2-^] absorbance ratio was used as an indicator of the extent of demineralization of dentin because of carious activity of the biofilm |
| **Appropriate Statistical methods** | **Statistical Analysis:**   - Shapiro-Wilk test for a normal distribution - The t test was used to compare pH values of biofilms, ratios of demineralised-to-sound dentine microhardness, Ca and P weight percentages, Ca/P ratios and log [amide I: HPO_4_^2-^] ratio between the test and control groups at the same lesion depth |
| **Test Organisms** | **Test microbes:**   - Streptococcus mutans ATCC (American Type Culture Collection) 35668 - Actinomyces naeslundii*ATCC 12014* |
| **Outcomes** | **Outcomes:**   - Biofilm count were reduced in SDF group than control (*p*<0.01) - After SDF application, surfaces of carious lesion were harder compared to control (*p*<0.05) - Both **Ca and P weight** percentages were higher after SDF application at 25µm but not the other measured depths only in blocks with *S. mutans* biofilms than control (*p*<0.05). No significant difference was found between SDF and control of dentine lesion by A. naeslundii - Lesion showed significantly reduced level of matrix to phosphate after SDF treatment (*p*<0.05) - **Time points:** Assessment after 7 days of incubation |
| **Notes** | - *In vitro* study, third permanent molar dentine blocks - Investigated SDF effect on dual species **(*S. mutans and A.* naeslundii)** biofilm - Evaluated biofilm count of ***S. mutans and A.* naeslundii** after treatment with SDF solution - Assessed viability and physiological activity of bioﬁlms using Confocal laser scanning microscopy (CLSM) - Assessed micro hardness and changes in mineral content of the carious lesion |

**Chu 2012**

| **Title:** **Effects of silver diamine fluoride on dentine carious lesions induced by *Streptococcus mutans* and *Actinomyces naeslundii*** | | |
| --- | --- | --- |
|  | **Risk of bias** | **Supporting reference and comments** |
| **Selection bias** |  |  |
| Baseline characteristics similarity/appropriate control selection | Low risk | QUOTE: " Extracted sound human third molars were collected. The teeth were stored in 1% sodium azide at 4^o^C. Thirty-two dentine blocks of 2×2×4 mm^3^ were prepared. The dentine blocks were examined under a stereomicroscope (×10 magnifications) to ensure they had no cracks, hypoplasia, or white spot lesions. Half of the surface of each block was coated with an acid-resistant nail varnish (Clarins, Paris, France) to serve as an internal control. The blocks were then sterilized with ethylene oxide.  A 300µL aliquot of each bacteria culture was mixed and inoculated on each dentine block sitting in a well of a 24-well plate with BMG. The plate was placed in an anaerobic chamber at 37oC for 3 days. The medium was refreshed daily without disturbing dentine surface. "  COMMENT: Complete detail of test and control selection and specimen preparation is given |
| Allocation concealment | Unclear risk | Not reported  COMMENT: Probably done |
| Randomization | Low risk | QUOTE: "Sixteen dentine blocks were underwent topical application of a commercially available 38% SDF solution (Saforide; Toyo Seiyaku Kasei Co. Ltd., Osaka, Japan) on exposed surfaces with a gravimetric micro-brush according to manufacturer´s instruction. The other 16 blocks were treated with water as a control".  COMMENT: Probably done |
| **Performance bias** |  |  |
| Blinding of Researchers | Unclear risk | Not reported  COMMENT: Probably done |
| **Detection bias** |  |  |
| Blinding of outcome assessors | Unclear risk | Not reported  COMMENT: Probably done |
| **Reporting bias** |  |  |
| Selective outcome reporting | Low risk | COMMENT: All expected outcomes reported |
| **Confounding bias** |  |  |
| Account for confounding variable | Unclear risk | Not reported  COMMENT: Probably done |

**Mei 2013(b)**

| **Title** | **Caries arresting effect of silver diamine fluoride on dentine carious lesion with S. mutans and L. acidophilus dual-species cariogenic biofilm** |
| --- | --- |
| **Material and Methods** | **Design:** *in vitro study*  **Setting:** *in vitro study* using human dentin blocks  **Country:** Hong Kong, China**.**  **Funding sources:** UGC General Research Fund (HKU765111M) Hong Kong University |
| **Materials** | **Specimen preparation:** Thirty dentin blocks (2x2x4mm^3^) prepared from extracted sound human third molars   - **Inclusion criteria**: extracted sound third molars stored in 1% sodium azide at 4^0^C - **Exclusion criteria:** Observation of dentin blocks under stereomicroscope (x10 magnification) exhibiting cracks, hypoplasia or white spot lesion were excluded - The surfaces of dentine blocks were polished by micro-fine 1,200 sanding paper under water - To eliminate the smear layer on the surfaces, dentin blocks were treated with 1% citric acid for 5 min and then rinsed with distilled water - Half of the surface of each block was coated with an acid-resistant nail varnish to serve as an internal control - The blocks were then sterilized with ethylene oxide for 16 hours   **Dual-species cariogenic biofilm (S. mutans and L. acidophilus)**   - Cultured on blood agar plates at 37^o^C for 2 days an aerobically - A single colony from each plate was picked to prepare 24-hour broth cultures in basal medium supplemented with 5% glucose BMG medium at 37^o^C under anaerobic conditions   **Inoculation of dentin discs with S. mutans and L. acidophilus**   - A 300µL aliquot (3x10^9^ cells/ml) of each bacteria culture was mixed and inoculated on each dentine block, placed in a well of 24-well plate BMG, in an anaerobic chamber at 37^o^C for 3 days. - The medium was refreshed daily, creating dentine lesion about 80 µm in depth - Dentin blocks were divided into two equal groups (test and control) - Of thirty human dentin blocks:   **Test group: 15** Dentin blocks assigned in the test group, were treated with 0.22mg±0.07 mg of 38% SDF (8.8µg±2.8µg fluoride) topically for 5 seconds on each block. And returned to the 24-well plate immediately with BMG.  **Control group: 15** Dentin blocks assigned in the control group were treated with sterile distilled water topically  **Evaluation after 7 days:**   - Test and control blocks were evaluated after 7 day-period after allowing the bacteria to grow and mature into biofilm |
| **Methods** | **Biofilm Assessment**  **CFU counting:**   - For specific identification of individual species, selective media plates were used   Mitis Salivarius agar plate for Mutans Streptococci identification  Rogosa agar plate for Lactocacillus acidophilus identification  **pH measurement:** pH test paper used to measure the resting pH of the biofilm  **Dentin block Assessment**  **Surface topography of the biofilm (Confocal laser scanning microscopy-CLSM):** to study viability and distribution of the biofilm   - To denote the ratio of dead-to-live bacteria, the bacteria on the dentin surfaces were labeled in situ using two fluorescent probes: propodium iodide and SYTO-9 dye - Five cellular images of each biofilm obtained using Confocal laser scanning microscopy (CLSM) and analyzed using an image analysis software (Image J)   **Surface topography of the biofilm (Scanning electron microscopy-SEM):**  ***X-ray diffraction analysis*:** To analyse crystal characteristics of the carious lesion  ***Evaluation- potential changes in the organic structure of the carious lesions (FTIR analysis:***   - Calculations were made from the spectrally derived matrix-to-mineral ratio (the ratio of the integrated area of protein amide I absorbance from 1585 to 1720 cm^-1^ to that of phosphate [HPO_4_^2-^] absorbance from 900 to 1200 cm^-1^)   ***Immunochemistry:*** To evaluate the quantity of intact collagen fibrils in the surface of carious lesion |
| **Appropriate Statistical methods** | **Sample size determination:**   - Not reported   **Statistical Analysis:**   - Parametric t test was used to compare log CFU, log [amide I: HPO_4_^2-^] ratio, and number of gold particles between SDF treated (test) and control groups at the same lesion depth |
| **Test Organisms** | **Test microbes:**   - Streptococcus mutans ATCC (American Type Culture Collection) 35668 - Lactobacillus acidophilus*ATCC 9224* |
| **Outcomes** | **Outcomes:**   - The log CFU of S. mutans and L. acidophilus in the test group was significantly lower than control group - The dead to live ratios from CLSM images indicates significant strength of antimicrobial effect of topical SDF application - SEM showed confluent biofilm in control group compared to test group   - X-ray Diffraction demonstrated the loss of crystallinity of dentine in test group was less due to the dissolution of hydroxyapatite crystal structure than in control group   - FTIR showed that log [Amide I: HPO42-] for test vs. control group was 0.31±0.10 vs. 0.57±0.13 (p<0.05)   - The gold labeling density in test vs. control group was 8.54±2.44/µm2 vs. 12.91±4.24/µm2 (p=0.04) - **Time points:** Assessment of biofilm after after 7 days |
| **Notes** | - *In vitro* study, third permanent molar dentine blocks used**,** - Investigated SDF effect on dual species **(*S. mutans and L. acidophilus*)** biofilm - Evaluated log CFU of ***S. mutans and L. acidophilus*** after treatment with SDF - Assessed viability and physiological activity of bioﬁlms using Confocal laser scanning microscopy (CLSM) - Assessed dentine structure changes using Electrochemical methods (XRD, FTIR and immune-labeling). |

**Mei 2013(b)**

| **Title:** **Caries arresting effect of silver diamine fluoride on dentine carious lesion with S. mutans and L. acidophilus dual-species cariogenic biofilm** | | |
| --- | --- | --- |
|  | **Risk of bias** | **Supporting reference and comments** |
| **Selection bias** |  |  |
| Baseline characteristics similarity/appropriate control selection | Low risk | QUOTE: " Extracted sound human third molars were collected. The teeth were stored in 1% sodium azide at 4^o^C. Thirty dentine blocks of 2×2×4 mm3 were prepared. The dentine blocks were examined under a stereomicroscope (×10 magnifications) to ensure they had no cracks, hypoplasia, or white spot lesions. The surfaces of dentine blocks were polished by micro-fine 1,200 sanding paper under water using Ecomet® 6 grinder- polisher (Buehlar, Waukegan, USA). The dentine blocks were treated with 1% citric acid for 5 min to eliminate the smear layer on the surfaces, and they were then rinsed with distilled water. Half of the surface of each block was coated with an acid-resistant nail varnish (Clarins, Paris, France) to serve as an internal control. The blocks were then sterilized with ethylene oxide.  A 300µL aliquot of each bacteria culture was mixed and inoculated on each dentine block sitting in a well of a 24-well plate with BMG. The plate was placed in an anaerobic chamber at 37oC for 3 days. The medium was refreshed daily without disturbing dentine surface. This generated dentine lesion about 80 µm in depth"  COMMENT: Complete detail of test and control selection and specimen preparation is given |
| Allocation concealment | Unclear risk | Not reported  COMMENT: Probably done |
| Randomization | Low risk | QUOTE: "Fifteen dentine blocks were underwent topical application of a commercially available 38% SDF solution (Saforide; Toyo Seiyaku Kasei Co. Ltd., Osaka, Japan) on exposed surfaces with a gravimetric micro-brush according to manufacturer´s instruction. The other 15 blocks were treated with distilled water as a control".  COMMENT: Probably done |
| **Performance bias** |  |  |
| Blinding of Researchers | Unclear risk | Not reported  COMMENT: Probably done |
| **Detection bias** |  |  |
| Blinding of outcome assessors | Unclear risk | Not reported  COMMENT: Probably done |
| **Reporting bias** |  |  |
| Selective outcome reporting | Low risk | COMMENT: All expected outcomes reported |
| **Confounding bias** |  |  |
| Account for confounding variable | Unclear risk | Not reported  COMMENT: Probably done |

**Hamama 2015**

| **Title** | **Effect of silver diamine ﬂuoride and potassium iodide on residual bacteria in dentinal tubules** |
| --- | --- |
| **Material and Methods** | **Design:** *in vitro study*  **Setting:** *in vitro study* using human dentin blocks  **Country:** Hong Kong, China**.**  **Funding sources:** Support of SDI Ltd (Bayswater, VIC, Australia) for donating the Riva Star agent to conduct the study |
| **Materials** | **Specimen preparation:** Forty-five, 2mm thick coronal dentin discs prepared from sound maxillary premolars, stored in 0.5% chloramine T solution at 4^0^C   - **Inclusion criteria**: sound maxillary premolars, extracted during past six months - **Exclusion criteria:** Observation of teeth under light stereomicroscopy exhibiting cracks were excluded - Dentine discs were prepared using a slow speed water-cooled diamond saw - To remove smear layer, the discs were cleaned with 5.25% sodium hypochlorite and 6% citric acid solution for 4 minutes in an ultrasonic cleaner - The discs were stabilized at the bottom of the 50ml centrifugation tube - The disc stabilization procedure was done inside an ultraviolet (UV) cabinet under short wave UV light (254nm) - The discs then further exposed to UV light for 1 hour to kill any contaminating bacteria after stabilization - The discs were then hermetically sealed to prevent contamination   **Inoculation of dentin discs with Streptococcus mutans:**   - *S. mutans* were isolated from human carious lesion and suspended in brain heart infusion (BHI) broth - Bacterial suspension was standardized to 3x10^6^ CFU/ml - 500 micro liters of bacterial suspension added to each tube and centrifuged twice at 1400g, 2000g, 3600g and 5600g respectively for 5 mins each, - Bacterial suspension was replaced with a fresh 500µl of the solution at the end of each cycle - Tubes were incubated for 24 hours at 37^0^C, prior to removal of the discs from the tube before dentin disc treatment and staining - Dentin blocks were randomly divided into two nine groups (five discs per group) - Of **forty- five dentine blocks**: - Positive control (n=5) dentine discs were infected and left untreated - Negative control (n=5) contained non-infected sound dentine discs - Silver diamine fluoride/potassium iodide (SDF/KI) applied of dentine carious lesion on test group (n=5, dentin blocks) - NaOCl-based gel (Carisolv) applied of dentine carious lesion   (n=5, dentin blocks)   - Enzyme-based gel (Papacarie) applied of dentine carious lesion   (n=5, dentin blocks)   - NaOCl-based gel (Carisolv)+ SDF/KI applied of dentine carious lesion   (n=5, dentin blocks)   - Enzyme based gel (Papacarie) + SDF/KI applied of dentine carious lesion   (n=5, dentin blocks)   - CHX applied of dentine carious lesion   (n=5, dentin blocks)   - CHX + SDF/KI applied of dentine carious lesion   (n=5, dentin blocks)  **Evaluation after 5 minutes of dentine disc treatment with the treatment regimen** |
| **Methods** | **Characterization of the viability and physiological activity of bioﬁlms using Confocal laser scanning microscopy (CLSM)**   - Observations were performed using confocal laser scanning microscopy (CLSM) at ﬁve randomly selected sites for each disc - Each 3D-reconstructed photograph was then analysed using Bio lmage L software to calculate the red/green ﬂuorescence (dead/live) volume percentage, using color segmentation method for characterization of the viability and physiological activity of bioﬁlms |
| **Appropriate Statistical methods** | **Sample size determination:**   - Not reported   **Statistical Analysis:**   - The mean biomass area percentage of each specimen was calculated and then subjected to one-way analysis of variance (ANOVA) followed by Tukey’s Honestly Signiﬁcant Difference post hoc multiple comparison tests - The distribution of the percentages of both live and dead bacteria was then checked by Kolmogorov–Smirnov test, while the equality of variance assumptions was checked with the modiﬁed Levene test. |
| **Test Organisms** | **Test microbes:**   - Streptococcus mutans |
| **Outcomes** | **Outcomes:**   - SDF/KI exhibited a potent antibacterial effect, as represented by a signiﬁcantly higher percentage of dead bacteria, in comparison with Carisolv and Papacarie (p < 0.05) - The application of SDF/KI following chemomechanical (Carisolv and Papacarie) caries removel gels application on the carious lesion signiﬁcantly reduced the viability of intra-tubular bacteria (p < 0.05) |
| **Notes** | - *In vitro* study, sound maxillary premolars dentine blocks used - Investigated SDF/KI versus CMCR gel (Carisolv and Papacarie) and CHX solution effect on (*S. mutans*) biofilm - Assessed viability and physiological activity of bioﬁlms using Confocal laser scanning microscopy (CLSM) |

**Hamama 2015**

| **Title:** **Effect of silver diamine ﬂuoride and potassium iodide on residual bacteria in dentinal tubules** | | |
| --- | --- | --- |
|  | **Risk of bias** | **Supporting reference and comments** |
| **Selection bias** |  |  |
| Baseline characteristics similarity/appropriate control selection | Low risk | QUOTE: Forty-ﬁve caries-free maxillary premolars stored in 0.5% chloramine T solution at 4^0^C were used in this study within six months of extraction. All premolars were observed under light stereomicroscopy and teeth exhibiting cracks were excluded. The preparation, infection and observation methods used in the current study followed the non-invasive protocol for comparing the antibacterial effectiveness of different disinfectants used in radicular dentine. This method has recently been modiﬁed for evaluation of the antibacterial effect of CMCR agents in coronal dentine.  COMMENT: Satisfactory detail of test and control selection and specimen preparation is given |
| Allocation concealment | Unclear risk | Not reported  COMMENT: Probably done |
| Randomization | Low risk | QUOTE: Each group consists of 5 discs (n = 5) and observations were performed at ﬁve randomly selected sites of each disc; 25 observation site/group.  Not reported  COMMENT: Details were reported to make judgment of low bias regarding randomization |
| **Performance bias** |  |  |
| Blinding of Researchers | Unclear risk | Not reported  COMMENT: Probably done |
| **Detection bias** |  |  |
| Blinding of outcome assessors | Low risk | QUOTE: The CLSM micrographs of the negative and positive controls and SDF/KI groups represent the baseline observations of this study (Fig. 1). The CLSM micrographs revealed that most of the intra-tubular bacteria were alive after a 5-minute dentine treatment with a NaOCl-based gel and in the positive control (infected non-treated) group.  COMMENT: Reported CLSM micrographs representing results of this study can rule out detection bias |
| **Reporting bias** |  |  |
| Selective outcome reporting | Low risk | COMMENT: All expected outcomes reported |
| **Confounding bias** |  |  |
| Account for confounding variable | Unclear risk | Not reported  COMMENT: Probably done |
